# Nature-based approaches: a mixed methods study of facilitators and barriers to implementation in CAMHS

**DOI:** 10.1186/s12913-024-11541-8

**Published:** 2024-11-08

**Authors:** Siobhan B. Mitchell, Beth Chapman, Rachel Hayes, Hélène Bonnici, Hazel Banks, Silvana Mareva, Rebecca Hardwick

**Affiliations:** 1 NIHR Applied Research Collaboration South West Peninsula, University of Exeter, South Cloisters, St. Luke’s Campus, Heavitree Road, Exeter, UK; 2 Cornwall Partnership NHS Foundation Trust, Cornwall, UK; 3 NIHR Applied Research Collaboration South West Peninsula, University of Plymouth, Plymouth, UK; 4 Faculty of Health and Life Sciences, University of Exeter, Exeter, UK

**Keywords:** Nature-based approaches, Children and young people, Mental health services, Implementation, Barriers, Facilitators

## Abstract

**Background:**

There is growing evidence that spending time with or in nature can be beneficial for health and wellbeing. Emerging evidence suggests potential benefits for staff and service users in healthcare settings, yet little is known about how to put Nature-based approaches (NBAs) into practice within the Child and Adolescent Mental Health Services (CAMHS) setting. The CAMHS Goes Wild project in Southwest England aimed to explore the implementation of NBAs within CAMHS, examining staff attitudes and understanding to identify potential benefits and challenges through a mixed methods study.

**Methods:**

The study involved three phases of data collection: an online survey with two waves, the first wave prior to the training, and the second four months post-training, and semi-structured interviews. Data collection was designed to fit around NatureWell training, attended by sixty-four CAMHS staff, which took place alongside the study. All participants were sampled from one NHS Trust and the study was open to both those who had attended the NatureWell training and those who had not. Data were synthesised to produce an understanding of staff attitudes towards NBAs and perceived barriers and facilitators to the implementation of this approach.

**Results:**

Ninety-seven staff responded to the wave 1 survey and 57 responded to the wave 2 survey. Fourteen staff members were interviewed. Data synthesis generated three themes: Tension between the culture of CAMHS and NBAs (Theme 1) and the need for buy-in and governance support (Theme 2). Theme three described the potential benefits of NBAs for staff and service users in CAMHS and is presented in a separate paper. The first two themes are presented in this paper.

**Conclusions:**

The implementation of NBAs in mental health service settings for CYP presents both significant challenges and opportunities. Our findings suggest multiple barriers to implementation, often in the form of organisational or cultural factors, such as the risk averse nature of the service. Our work also elucidates several potential facilitators which may address or mitigate some of these barriers. These potential enablers, such as harnessing the role of firsthand experience, warrant further exploration in the implementation of NBAs in CAMHS.

**Supplementary Information:**

The online version contains supplementary material available at 10.1186/s12913-024-11541-8.

## Background

There is growing evidence that spending time with or in nature can be beneficial for health and wellbeing [1–3]. Nature-based approaches (NBAs) is an umbrella term which refers to activities which promote direct involvement with the natural environment, increasing contact or connection with nature, either through going outdoors e.g., urban nature, woodlands, blue spaces, or bringing nature indoors. Nature-based interventions (NBIs) for health refer to NBAs with a specific holistic health goal in mind. The concept of “nature connectedness,” an individual’s subjective sense of connection to nature, is more strongly linked to wellbeing than mere exposure to nature [4]. This connection also encourages pro-environmental behaviours, crucial during climate and health crises [5].

Existing research by Hunt et al. (2022) suggests that NBAs can benefit inpatients and staff within Child and Adolescent Mental Health Services (CAMHS).; engaging with NBAs helped patient’s emotional regulation, enhanced the therapeutic relationship between staff and patients, and supported long-term recovery by connecting patients with activities that can be sustained beyond discharge [6]. Staff also benefited, stating it helped with burnout and supported stronger peer relationships [6]. Community CAMHS consultations typically take place indoors, limiting access to nature for staff and patients. However, exposure to natural environments could enhance mental health for both groups. Research conducted in non-healthcare contexts suggests nature contact boosts mental health, cognitive ability, and reduces stress and health complaints [7–9]. In the United Kingdom’s National Health Service (NHS), where services are under increasing stress [10], there has been a large-scale emphasis on staff wellbeing teams who promote work-life balance and health checks [11]; while it is beyond their remit to influence workload and capacity, advocating simple changes within the work environment could be valuable. Exposure, experience, and context can influence individual state changes in nature connectedness and therefore, encouraging nature connectedness amongst employees could improve staff wellbeing and productivity and reduce sickness levels [12, 13]. In CAMHS, NBAs would encompass using nature within practice that increases either contact or connection with nature. This could include bringing nature in, such as plants, objects, photos; or going outdoors. Nature could provide a change in context from which to deliver the intervention e.g., walk and talk; exist as a co-therapist e.g., using metaphor within nature; or be directly embedded within the intervention to facilitate a specific holistic health outcome [4]. While evidence around the use of similar approaches with children and young people is currently lacking, offering services outside traditional clinic settings may benefit their wellbeing and provide insights into more age-appropriate, personalised care.

While evidence points to the benefits of implementing NBAs, little is known about how to put this into practice within the CAMHS setting. The challenges of implementing new interventions or practices within CAMHS have been documented; with a relatively low uptake of new evidence-based practices or interventions into clinical practice, meaning these interventions do not always reach those in need [14]. A systematic review of barriers to implementation identified several challenges for the implementation of evidence-based practices in CAMHS, including clinician attitudes and flexibility, poor access to resources, and lack of access to funding [14]. Implementation facilitators within CAMHS included targeted funding and access to resources, supportive staff, and leadership committed to innovation and skills [14]. Other studies exploring implementation challenges within this context highlighted the background of staff as key in terms of how or whether particular treatment approaches are adopted; the impact of stretched service capacity upon treatment choices; and lack of time to explore alternative approaches [15, 16]. For CAMHS staff in schools, practical issues such as flexibility, relationships with school staff, and logistical constraints (e.g., timetables, space) complicate implementation [17]. Moreover, NBAs may pose greater implementation challenges due to perception of greater resource need, and through necessitating a culture shift away from the traditional clinic-based model of delivery. Existing studies report barriers to implementation including policies around safety and risk, geographic accessibility, and staff attitude and team culture and ethos [6, 18].

There is growing recognition of the benefits of incorporating NBAs into mental health care services, yet there is wide variation in the implementation of these approaches, including the scale, language used, local availability and delivery of these interventions [19]. Acknowledging this variation and supporting local tailoring in the implementation can be essential to improve effectiveness [20]. Integrating NBAs into pre-existing healthcare services can present context-specific challenges. For example, the quality of NHS estates varies geographically and between services, some can be without ventilation or outdoor space. It is acknowledged that a systematic overhaul of NHS estates is unlikely, instead, the implementation of NBAs within existing practices and increasing opportunity for contact with nature at work could be promoted.

Studies by Hunt et al. (2022) and Tambyah et al. (2022) have explored the implementation of NBAs with young people in health service settings, identifying several key barriers and facilitators [6, 18]. Staff have highlighted the need for additional time and resources, noting that NBAs often require extra time for planning, travel, and managing increased risks associated with outdoor activities [6, 18]. Geographic accessibility poses another significant challenge, alongside organisational barriers such as health and safety policies and risk management protocols [6]. Furthermore, patient factors such as varying risk levels, clinical needs, and symptoms like anxiety or lack of motivation can hinder engagement [6]. Staff and patient scepticism towards the effectiveness of NBAs and the role of staff attitudes towards NBAs in the workplace were also noted as important [6, 14, 18]. Having a supportive workplace culture towards NBAs such as a shared team approach and feeling personal enthusiasm for NBAs are both factors that are reported to support effective implementation [14, 18].

Implementing NBAs within children and young people’s services can warrant additional considerations such as barriers around engagement, risk assessment, and parent buy-in/endorsement [6]. For example, the model of delivery utilised for NBAs may conflict with family expectations as to what a CAMHS service is and what it provides. Ways of mitigating these barriers and promoting better engagement have been put forward, such as employing the co-design of activities with young people and families to boost engagement and interest [6].

CAMHS Goes Wild is an innovation within a specialist CAMHS service within the Southwest of England exploring the utilisation of NBAs within its delivery. The CAMHS Goes Wild project explored attitudes and understanding of NBAs amongst CAMHS staff to better understand potential benefits and challenges of adoption and implementation of this approach within CAMHS.

## Methods

Data are drawn from a mixed methods study looking at NBAs in CAMHS. Interview and survey data are presented. Ethical approval was obtained from the Health Research Authority (23/HRA/0191). Written informed consent to participate in the survey and interviews was obtained from all participants.

### Design

Two introductory training away days were offered to all CAMHS staff in the Trust, with 64 attending in total. This was co-facilitated by CAMHS clinician leads for this project and the Natural Academy. Subsequently, a higher level six-day accredited facilitator training course was provided, with 16 staff attending. For those who attended the six-day training it was not mandatory to have attended the introductory away day, some attended both and others attended only the six-day training. Training for using the NatureWell approach was based around the five pathways to nature connectedness: contact, beauty, emotion, compassion, meaning. Training courses were independent of the research study. The Natural Academy is a not-for-profit eco-social enterprise offering accredited training course to people to enhance their work with people in nature. A variety of training is offered which imparts the NatureWell approach such as the aforementioned NatureWell facilitator training, which was designed for those working in health and social care settings to upskill them to deliver nature-based services.

**Fig. 1:**
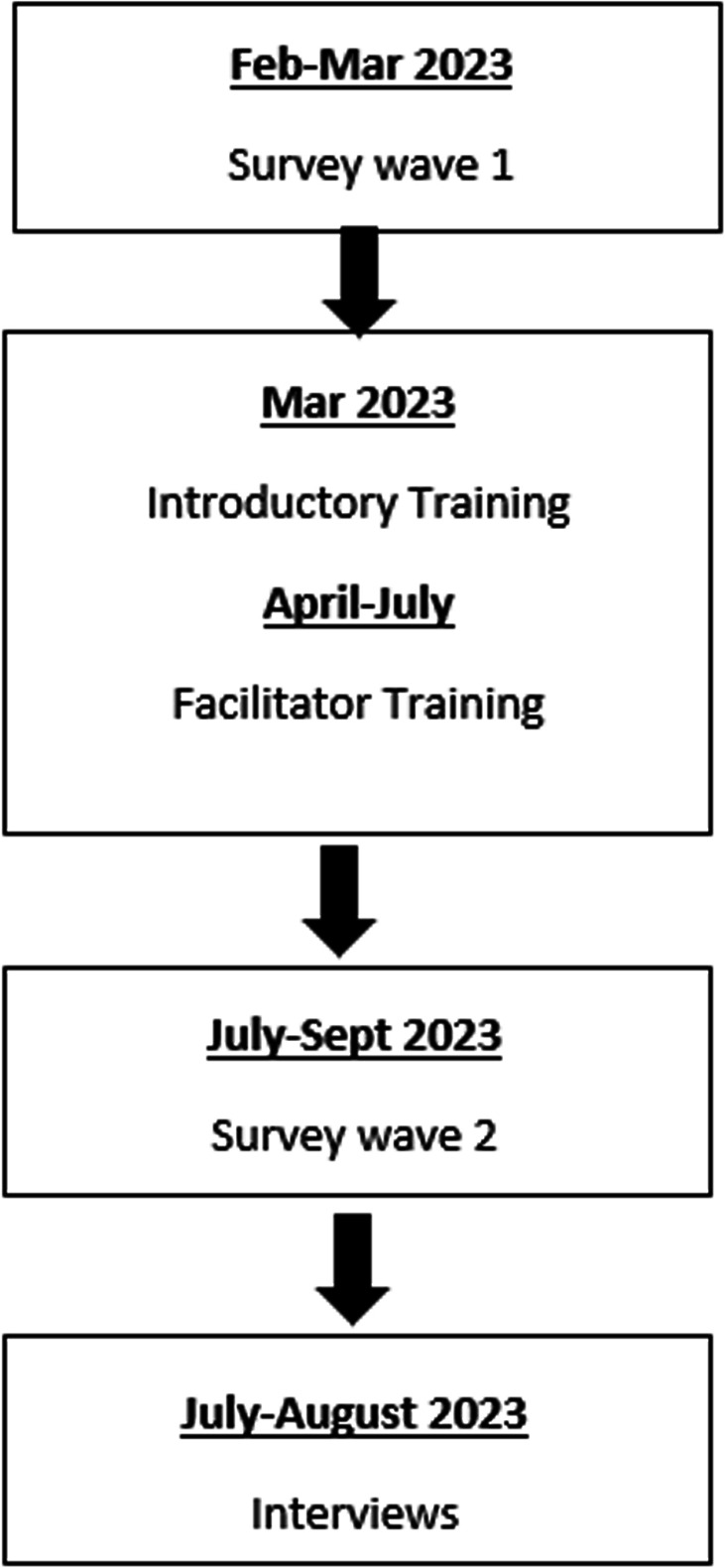
Study design flow chart

Data were collected in three consecutive phases (see Fig. 1), following a sequential explanatory design. First, an online mixed-methods survey captured participants’ quantitative and qualitative knowledge and attitudes at wave 1 (Feb-March 2023) prior to the Natural Academy training. A follow-up survey measured participants’ responses to the same questions four months later (wave 2), after the Natural Academy training. Collecting data at these two timepoints allowed the researchers to observe any differences or consistencies pre and post the Natural Academy training. Comparative analysis of the data was used to inform the next phase of data collection. The third phase of the study involved in-depth qualitative data via interviews with 14 participants. The outcomes from phases one and two were used to design the interview questions. A realist informed approach was adopted to help identify the potential mechanisms generating the outcomes that were originally observed in the survey data [21, 22]. Synthesis of data from all phases of the study was carried out to offer a comprehensive response to the research questions. Specifically, data were synthesised to produce an understanding of staff attitudes towards NBAs and perceived barriers and facilitators to the implementation of this approach. A process of triangulation was used [23]. After analysis, the data were combined; and the original themes were integrated, reviewed and refined. This produced a final set of themes with corresponding quantitative data.

### Sampling and recruitment

All CAMHS staff working at one NHS Trust in the South of England were invited to participate in a short online survey administered through Qualtrics. The survey was open to all staff, regardless of their attendance at any training, this was intended to provide us with an understanding of a breadth of attitudes across the service.

For interviews, convenience sampling was employed. All CAMHS staff who completed the online surveys were invited to indicate if they would be happy to be contacted for an interview to explore the use of NBAs in their work. Those interviewed were from a range of roles within CAMHS (See Table 1), worked across clinical, home, and school settings and with a range of patient groups. Some interview participants (*n* = 8) had undertaken training with the Natural Academy as part of the CAMHS Goes Wild project and some (*n* = 6) had not. Of those who had completed the training, five of these had completed the introductory training only, and three had completed the facilitator training.

**Table 1:** Interview participant demographics

*N*	14
Role	Clinical associate psychologist: *N* = 5, Education mental health practitioner: *N* = 4, Clinical psychologist: *N* = 1, Family therapist: *N* = 1, Nurse: *N* = 1, Assistant psychologist: *N* = 1, Not specified, *N* = 1.
Context	Clinical: *N* = 9, School-based: *N* = 3, Mixed setting (i.e., school/home/clinic): *N* = 2
Level of experience	Management/Senior: *N* = 4, Mid-career: *N* = 4, Junior/Trainee: *N* = 5, Not specified: *N* = 1
Attendance at training	Both phases of training (introductory and facilitator training), *N* = 3; phase 1 (introductory training) only, *N* = 5; no training, *N* = 6.

The rationale for sampling both those who had done the training and those who had not was that this would enable us to understand a range of barriers to implementation, including barriers to attending the training itself. Respondents were offered a £5 bank transfer, shopping voucher or charity donation as compensation for their time.

### Staff Survey

The survey asked questions about attendance at recent away days and facilitator training, and staff experience and attitudes towards NBAs. The survey also used validated measures to assess nature connectedness, burnout and wellbeing, which are reported elsewhere [10].

### Survey measures

#### Knowledge and attitudes towards nature-based interventions

Four unvalidated bespoke questions assessing staff knowledge and attitudes toward nature-based interventions were included. Two questions had a binary response style (Yes/No): “I am aware of NHS services offering Nature-Based Interventions to children and young people” and “I have delivered Nature-Based Interventions to children and young people”. The remaining two questions had a 5-point scale response style ranging from “Strongly Agree” to “Strongly Disagree”: “I am confident in delivering Nature-Based Interventions” and “I would like to be able to deliver Nature-Based Interventions”.

#### Free-text questions

Respondents were also presented with two free-text questions: ‘Can you think of any benefits to using nature-based interventions in your work?’ and ‘Do you have any concerns about using nature-based interventions in your work?’.

### Analysis

Data analysis explored staff willingness to use NBAs; interest in and awareness of NBA use in their workplace; experience of delivering NBAs to children and young people (CYP); and confidence in delivering NBAs. Data were analysed descriptively to address each area and to explore changes from wave 1 and wave 2. All analyses were conducted in R version 4.2.0 using psych package version 2.3.3. Free text responses were extracted from the wave 1 and wave 2 questionnaire data and analysed together. Responses were read and grouped thematically by one of the co-authors according to key words or concepts.

### Staff interviews

#### Interview procedure

All participants provided written informed consent, including for audio-recording of the interviews. Semi-structured interviews took place between July and August 2023, by video call. All interviews were digitally voice-recorded and transcribed verbatim. Each recruited participant was assigned a unique identifier code.

Topic guides covered staff experience and attitudes towards integrating NBAs in CAMHS. The topic guides were developed following analysis of the survey data and were informed by a realist approach, because we were interested in understanding whether changes had occurred, and if so, what had caused that, how and why. Interviews were theory-driven with questions framed around understanding perceptions of the outcomes for staff and service users (e.g., What do you consider to be the outcomes or changes that using NBAs has had/may have for staff? ), how outcomes may differ for different people (e.g., Do you think that the outcomes have been the same for all staff? In what ways have they been different? ), potential mechanisms (e.g., We are very curious about how NBAs cause outcomes. How do you think the programme has caused, or helped to cause [one outcome identified by respondent]? ), how mechanisms may differ by person or place (e.g., there are lots of ideas about how NBAs work, and we think they probably work differently in different places or for different people. One of those ideas is it gives staff more autonomy to be creative. Has it worked at all like that here or for you? Can you give an example? ) and barriers or facilitators for implementation of NBAs (e.g., We’ve heard that NBA work differently in different places, what do you think it is about this place that makes it work so well/less well? ). For full topic guide see Appendix 1.

### Analysis

Interview recordings and transcriptions were stored on an encrypted hard drive. Once transcribed, interview data were managed using QSR International’s NVivo12 qualitative data analysis software (QSR International Pty Ltd., 2012) and were stored securely and password protected.

Framework analysis was employed [24]. The first stage of analysis, completed by SM, BC, HB, and RH, started with ‘indexing’ a small sample of interviews, to gather an insight and overview of the data. A thematic framework was then created, building on the initial review of data. This framework identified key concepts and was used to code all remaining interview data [24]. The next stage involved writing summaries of each interview for every code. This allowed for comparison, exploration and explanation of patterns [25]. This method facilitates systematic and transparent data analysis, and enables researchers to identify patterns or commonalities, as well as contradictions in and between participants’ accounts, so they can explore and test explanations for those patterns [25]. Refined themes were reviewed by BC, SM and HB, and final themes and subthemes were then agreed [26].

### Rigour

Throughout the data analysis process consideration was given to trustworthiness, specifically the credibility, dependability and transferability of the data. A reflexive approach was adopted to reduce bias and enhance credibility [27]. Thoughts on how to interpret the data were noted and regular meetings of the research team provided space to reflect on and discuss these interpretations. This process involved documenting decisions made during the research process and creating an audit trail to ensure transparency and strengthen the dependability of the study. This active construction of interpretations is important in reflexive research; where it is acknowledged that researchers simultaneously construct interpretations and question how those interpretations came about [28]. The differing roles of the research team with regard to their involvement in the implementation of NBAs in CAMHS (BC is directly involved in implementation, all other authors work in a research only capacity), meant that reflexivity was particularly important, acknowledging the experiences of the team to ensure that the themes generated were grounded in the evidence presented in the transcripts. The mixed methods study design enabled us to use triangulation, employing multiple data sources to cross-verify findings and strengthen the credibility of the analysis. We carefully considered and outlined our sampling strategy to allow transparency and clarity concerning the transferability of our findings.

## Results

### Survey

Demographic data for survey respondents for wave 1 and wave 2 are presented in Table 2. Data are presented for those who responded at each timepoint, with a 59% retention rate.

**Table 2:** Survey respondent demographics

	Wave 1	Wave 2
Gender	97 (83f, 13m, 1 gender fluid)	57 (49f, 7m, 1 gender fluid)
Work pattern	59 full-time, 29 part-time (md: 9)	36 full-time, 17 part-time (md:4)
Role	Other clinical: *N* = 36, Clinical associate psychologist: *N* = 16, Nurse: *N* = 12, Non-clinical: *N* = 12, Doctor: *N* = 8, Clinical Psychologist: *N* = 5, Psychology intern: *N* = 3, Other (occupational therapy, health and social care, etc.: *N* = 5).	Other clinical: *N* = 24, Clinical associate psychologist: *N* = 8, Nurse: *N* = 7, Non-clinical: *N* = 6, Doctor: *N* = 5, Clinical Psychologist: *N* = 3, Other: *N* = 4.
Age	Under 25, *N* = 8; Between 25 and 34, *N* = 27; Between 35 and 44, *N* = 25; Between 45 and 54, *N* = 28; Between 55 and 64, *N* = 7; 65 and over, *N* = 2.	Under 25, *N* = 4; Between 25 and 34, *N* = 15; Between 35 and 44, *N* = 16; Between 45 and 54, *N* = 19; Between 55 and 64, *N* = 2; 65 and over, *N* = 1.
Time at Trust	Less than 1 year, *N* = 36; Between 1 and 2 years, *N* = 6; Between 2 and 5 years, *N* = 34; Between 5 and 10 years, *N* = 12; Between 10 and 15 years, *N* = 5; Over 15 years, *N* = 4.	Less than 1 year, *N* = 22; Between 2 and 5 years, *N* = 27; Between 5 and 10 years, *N* = 5; Over 15 years, *N* = 3.
Attendance at training	Both phases of training (introductory and facilitator training), *N* = 10; phase 1 (introductory training) only, *N* = 38; no training, *N* = 49.	Both phases of training (introductory and facilitator training), *N* = 4; phase 1 (introductory training) only, *N* = 22; no training, *N* = 31.

For the free text section of the survey, of the 97 Wave 1 respondents, 47 responded to the free-text question “Do you have any concerns about using nature-based interventions in your work?” 25 respondents to the survey did not have any concerns and 25 respondents left this blank. Of those who did not have any concerns or left this blank, 28 completed wave 2 questionnaires and 13 of those described concerns the second time around. Four main concerns were identified: (i) Resource; (ii) Operational issues; (iii) Current culture; (iv) Impact on delivery of therapeutic intervention.

### Interview

Fourteen interviews were conducted. Demographics of staff interviewed are detailed in Table 1. Interviews ranged from 30 to 52 min, with an average duration of 42 min.

**Fig. 2:**
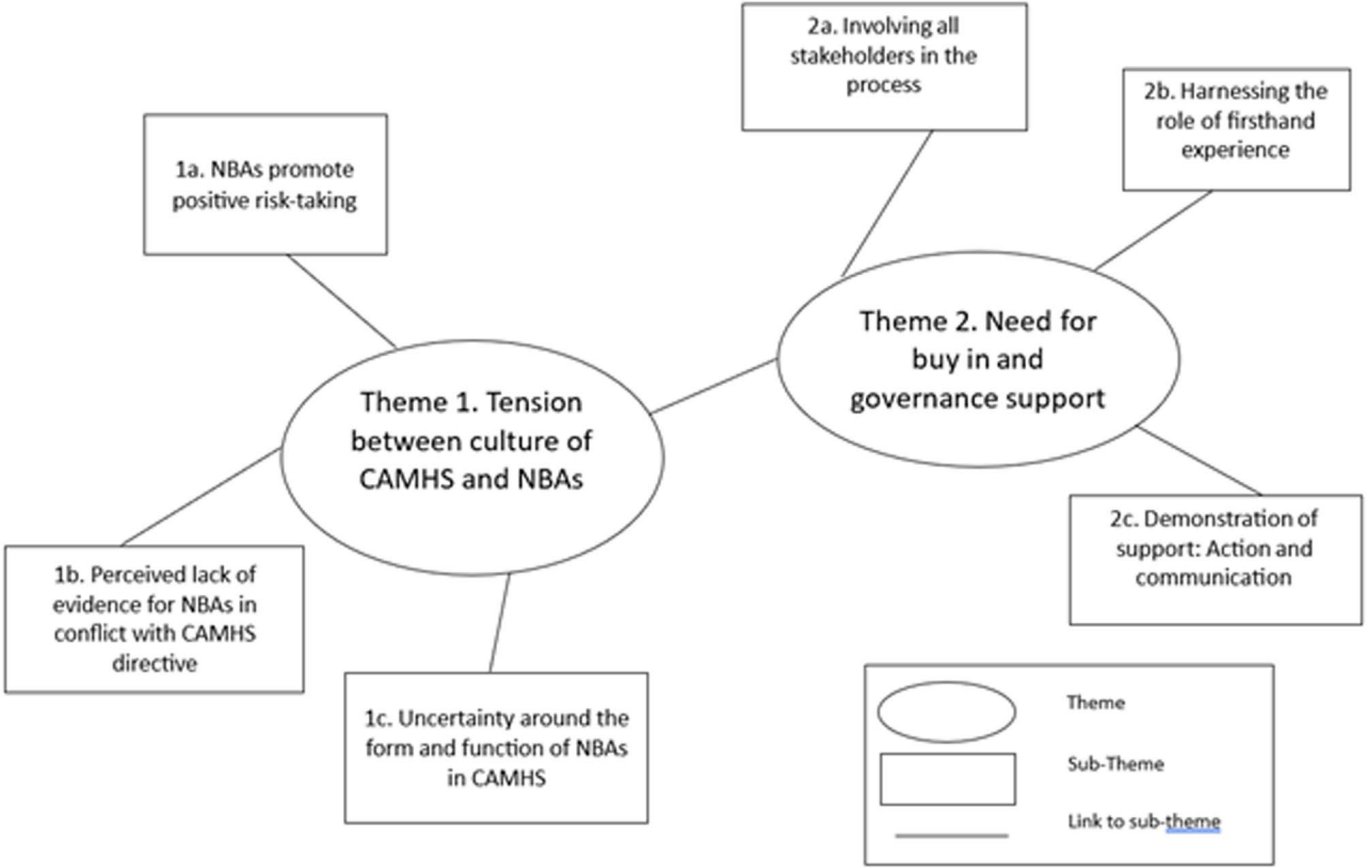
Thematic Map

Our synthesis of survey and interview data is illustrated by three themes. The data evidence a tension between the culture of CAMHS and NBAs as a core barrier to the implementation of this approach (Theme 1) and the need for buy-in and governance support as a potential enabler of NBAs in this context (Theme 2). Theme three described the potential benefits of NBAs for staff and service users in CAMHS. The first two themes are presented in this paper (see Fig. 2), the third theme is presented in a separate paper. Quotes are presented throughout from interview participants (P1-P14), and survey respondents (wave and participant number). The findings from themes 1 and 2 indicate that behind a shifting awareness of NBAs in the NHS, there are structural and cultural mechanisms that play a crucial role in supporting effective implementation.

### Theme 1: tension between culture of CAMHS and NBAs

Staff raised concerns about the culture of CAMHS in relation to the implementation of NBAs. Specifically, there were concerns about how using NBAs may be perceived by other staff and that there may be resistance and a lack of understanding of their value and validity i.e., NBAs not considered to be “proper work”.

I feel that the wider team need to understand its place in clinical work. I think sometimes it’s seen as ‘a galivant in the forest’ just because the practitioner fancies a break from “proper” work and that view needs to change. (Wave 2, P64)

It was identified that to deliver NBAs requires a culture shift both for the staff and for families whose expectations may be that care will be delivered within a clinic-based model: “How can we ‘sell’ it to families who have high expectations about wanting something more ‘clinical’?” (Wave 2, P32). There was also concern about how this approach would fit within a culture that is currently “…risk averse, conservative and blaming” (Wave 2, P82).

This tension is reflected in the quantitative results: despite a consistent and high desire to implement NBAs (W1:90%, W2:91%), staff reported relatively low levels of confidence to deliver them (W1:17%, W2:33%) and the majority had no experience delivering NBAs to CYP, which stayed consistent across both timepoints (W1:68%, W2:63%). This suggests a conflict between staff ambitions to implement NBAs and their ability to do so in the current context of CAMHS.

Staff also described conflict between implementing innovative, new approaches such as NBAs and delivering on ‘the basics’ with cultural and operational shifts needed to support adoption and implementation of this in practice.

I think it’s fitting it in with like I said what we’re commissioned to do, because we have got waiting lists, we’ve got huge pressure on services…it’s very hard to argue for innovation when what is sometimes seen as perhaps the basics not being done. We need to get the basics of treatment right before we can do all the bells and whistles. (P1)

I wonder if just the culture of our organisation…just shifting the culture to be more flexible and be outside, not just sit in a clinic room. I suppose it is a barrier, it’s not there intentionally being a barrier but I think it’s just change, isn’t it, organisations are scared of change. (P8)

Perceived conflict between the culture of CAMHS and NBAs also extended to staff wellbeing. Staff described CAMHS as a stressful workplace where they felt that their wellbeing is often not valued or prioritised. They reflected that while engaging in something like NBAs as part of work may also be beneficial for staff, this would feel challenging in an environment where staff wellbeing is not highly valued.

I think it [using NBAs] would definitely improve staff wellbeing. I think that would come with a level of guilt for clinicians. We’re used to feeling like we have to feel run down at the end of a day, and not feeling like that can feel like you haven’t contributed or sacrificed enough of yourself…The wellbeing improvement, I think staff would struggle to acknowledge that. (P2)

### Sub-theme a: NBAs promote positive risk-taking

Staff felt that adopting NBAs may require staff to step outside of the risk averse culture and to engage in more positive risk-taking. Staff described a tension between the risk averse nature of CAMHS as an organisation and what is required to implement NBAs; staff reflect that they would need to feel that there is permission to step outside of this and that it would require dynamic risk assessment and positive risk-taking: “Well, it would be a bit scary as well. I think it would be a bit scary for some clinicians. Some clinicians like yourself might go, “Ooh, you don’t do things like that”…It’s about culture.” (P7). This would feel challenging for some staff and would require support in terms of both organisational permission and tools/resources e.g., ways to assess and manage risk more dynamically.

It doesn’t feel like it’s part of the structure of the service it can feel quite intimidating as a member of staff, and you can feel like you’re sort of going a bit rogue, when really all you’re doing is adapting the environment. It can feel like you need a bit more back-up before you can justify doing that. (P2)

Staff described wanting more training on the use of NBAs to feel more confident and competent when incorporating them into clinical work: “How to effectively risk assess for individuals and what kinds of activities to deliver to ensure the intervention is meaningful and purposeful” (Wave 1, P77). This corresponds to the relatively low levels of confidence and experience in delivering NBAs reported in the quantitative findings.

In addition, respondents perceived potential risk to the delivery of the therapeutic intervention or assessment. Staff were concerned that maintaining confidentiality, privacy and therapeutic containment may be more difficult when based in nature and that it may be an unhelpful distraction: “I do a lot of trauma work, it wouldn’t be appropriate to do all my work in nature due to privacy concerns” (Wave 1, P40) and “My only concerns regarding nature-based work would be the issues around privacy and trying to find a space where the YP in which I would be working with felt comfortable and not distracted” (Wave 2 P63).

Those with experience in professions outside of CAMHS held more broad views on risk and its management which were more embracing of and aligned with the approach required for implementing NBAs.

I think it’s practitioner confidence in terms of safety…there’s lots of things that can happen outdoors that you need to be aware of… But just being able to have that confidence I think to have those conversations. I think there’s always that worry about what management are going to say but I think I feel quite skilled in being able to do risk benefit analysis because I’ve done that previously. (P5)

Those with more diverse experiences acknowledged risk as normal for adolescents more broadly and advocated less focus on this and more on connection: “There’s so much risk emphasis and not enough connection emphasis. Actually, adolescence is a risky time anyway. It’s a risky time no matter where you put an adolescent” (P7).

Those with greater experience or in more senior roles reflected that it was easier to engage in positive risk-taking.

I think I’m in quite a privileged position in my job role because I am trusted and respected that I know what I’m doing. If I’m going, “Yeah, I think the right thing right now is to go for a walk round the block with my client,” or, “I think we should do our first session in the garden,” people trust my clinical judgement… I think it could be more challenging for some more junior members of staff to argue that with managers that might feel a bit cautious, or supervisors that might feel a bit cautious about that. (P1)

Overall, staff described a need for consideration of, but not focus on, risk and the need for organisational support to enable the flexibility required to share or dynamically assess risk. Providing staff with clear messages, permission, and support around risk was described as essential to supporting flexible, creative, and innovative ways of working: “We receive mixed messages all the time “use nature-get outside and be flexible and creative” vs “not allowed to do anything flexible due to risk”” (Wave 1, P74) and “…more permission to go outside and be a bit more flexible and not be so worried about how are we going to risk assess going outside” (P8).

Staff described how a framework that includes risk assessment for patient, staff and the public would help staff to feel they have permission to work in this way and would support those who have not worked outside of clinical spaces to work more dynamically with risk.

I think for a lot of the worries a recognised proforma for risk for outdoor practice across the trust would be a really useful document…I don’t necessarily think that it’s 100% necessary as a thing. But I think the comfort that it would give to some, “Oh well, the trust have got these policies and if it goes wrong we will have the backing of the trust”…I think that would be of some comfort and allow people to be creative… (P11)

### Sub-theme b: perceived lack of evidence for NBAs in conflict with CAMHS directive

There was a desire to know more about the evidence base and an awareness that this is emerging: “I would like to know/ understand more about the approaches” (Wave 1, P33).

Staff perceived a lack of evidence for NBAs which was in conflict with the CAMHS directive to deliver evidence-based therapy.

…at the end of the day I need to do the job that I’m employed for, and that is to work with our most complex cases and to help them work towards wellness…the evidence-based treatment…that has got to be number one with what we’re doing…If we’re enhancing their nature connectedness that’s fantastic, but that’s not what we’re commissioned for. We have to be showing that we’re doing something effective when it comes to mental health, it’s got to be specific. (P1)

Greater evidence for NBAs was described as crucial to the motivation of both the wider organisation, staff and stakeholders, such as parents, to adopt and implement NBAs.

…I think part of the conversations that we’re having at MDT (multidisciplinary team) at the moment is that there’s the evidence behind nature-based interventions is limited… So if there were more research or evidence for nature-based interventions then there might be less reluctance from services to say, “Yes this is the offer from assessment.” (P13)

This drive for evidence was also described as important for empowering front-line staff to feel they had permission to work in this way and to be able to ‘back up’ their practice.

It would be a little bit like, when I think way back when we were trying to encourage parents to use Makaton signs with their kids, and we could show them as much as we liked and we could model it, but they never quite believed it until we could signpost them to somewhere that said, “Hey, the evidence says that this does have a positive effect on verbal communication.” They weren’t totally sold on it until we had that evidence and we could pass that onto them. That’s not just us, that’s a wider shift. (P3)

For many of those front-line staff interviewed, their personal experiences of nature formed part of their evidence in support of this approach: “Yeah, I mean 100% I really honestly, I mean it’s not a scientific approach in any way, but I just know personally when I’ve been out in nature during my working day I feel so much more relaxed” (P5).

These individuals valued experiential evidence highly and, in some cases, questioned the need for more empirical evidence in this space.

I know there’s a body of evidence but I almost don’t need to know that there’s a body of evidence because I know that it’s essential for me and I can see that in this young person that it’s something that she’s deeply connected to, that offers her support and all sorts of benefits. (P4)

How evidence-based does the practice have to be before it’s accepted by the more learned? …there’s some doctors with wonderful levels of experience, how evidence-based does it have to be? On the back of that, will a pursuit of evidence-based nature practice take away the beauty of mystery and uniqueness of every experience in nature? By creating an evidence-base and maybe even a nature pathway…are you then missing the point. (P11)

### Sub-theme c: uncertainty around the form and function of NBAs in CAMHS

A perceived lack of evidence was not just a barrier for initial adoption of NBAs, but also where they are adopted, how they are implemented. The lack of knowledge of evidence for the effectiveness of NBAs led some staff to see them more as an adjunct to treatment, rather than the intervention itself. The implication of this was that staff described using NBAs as an ‘add on’, something that was not embedded in their practice, therefore limiting the use of NBAs within this context: “I suppose my instant thought was where does this fit with their evidence-based pathways? Is this going to be an intervention in and of itself, or an adjunct to enable people to engage with evidence-based treatment?” (P1).

Some staff described using nature as tool in own right (e.g., connecting with nature as part of therapy): “Actually sometimes it’s a learning feature isn’t it for children, they talk about the rain technique, being outside and being able to tell them there’s uncomfortable weathers the same as uncomfortable feelings” (P5)

I think it’s that basically by using nature as a tool…I think just finding your way through nature and engaging with trees and leaves and dirt and sky and grass in a therapeutic way using that as a tool for healing is quite different than just sort of being in nature and doing the therapy anyway… (P6)

Others described how this may more likely take the form of treatment as usual but in nature (e.g., doing mindfulness or CBT outside in nature as opposed to in the clinic) either because they felt that this was where NBAs ‘fit’ or because of aforementioned pressures to focus on interventions they perceived to have a more established evidence base:

It was like a one-off for them. It wasn’t embedded in their practice. They regularly take kids for a walk up the hill to the monument above [local area] and they take them out to [local woodland] and so you could be doing those sorts of activities. (P7)

…there’s a lot of pressure on throughput at the moment in our service and it almost felt like well one people on our internal waiting list have been made an offer of an intervention, so if we would say, “Here’s this group that you could do,” it wouldn’t be, they would still be entitled to the original intervention. So it would be an addition rather than this is the intervention. (P13)

### Theme 2: need for buy-in and governance support

Quantitative results indicate a growing awareness among staff of NBAs being offered in the NHS (W1: 45% < W2:72%), along with an increase in confidence to deliver them (W1:17% < W2: 33%). However, the majority still had no experience delivering NBA’s to CYP (W1:68%, W2:63%), suggesting that greater support and awareness across a range of stakeholders is required to facilitate implementation.

The highest number of concerns in the free text survey were about operational challenges and the message of support that putting in place formal structures and processes would communicate: “We need the bosses to agree how to use our training” (Wave 2, P51). For example, many staff identified the need for a delivery framework to support with the implementation of NBAs: “…need to apply models to the work when just focusing on the surroundings…often more beneficial use of nature” (Wave 1, P3). This may need to incorporate additional policies or procedures and organisational support and governance was thought to be crucial. However, it was also raised that a “model” of delivery may be unnecessary and manualising and medicalising this can take away from the benefits of just being in nature. The concept of “permission” was recurrent.

### Sub-theme a: involving all stakeholders in the process

Staff described the importance of involving a variety of stakeholders, including senior management, service users, parents, schools, staff (practitioners and non-practitioners), in conversations about the adoption and implementation of NBAs. This was important on two levels: First, for senior management, greater buy-in could work to leverage more optimal conditions for staff to implement NBAs such as permissions, time, and resources; Second, getting those who use and deliver services ‘on board’ could help to better navigate any barriers to implementation by allowing them to have a say in shaping whether and how new interventions such as NBAs are put into practice, thereby making adoption more likely and implementation smoother.

It’s about how we do it, isn’t it? How do we get senior leaders on-board? But how do we also get people on the ground on-board? It’s got to be all levels that are for this as well as young people and families, so it’s a huge number of stakeholders involved to make this kind of shift. If we’re going to do it on a big scale. I think we can all do it on a little mini scale like when I go and say, “Right, I’m walking around the dock with my client,” or, “Yeah, we’re going to do our first session in the garden,” that’s one thing. But if we’re going to do this on a big way wholescale across CAMHS, that involves a lot of buy-in. (P1)

Staff described the need to get senior management ‘on board’ and that the ‘buy-in’ of other key stakeholders such as parents and schools, was crucial: “Do some pilots/trial runs of children seeing what they think, get their perspective. Talk to families. Just to make sure what we’re doing is actually going to be beneficial to the service users really”. (P9)

While many of the staff interviewed were keen to adopt NBAs, a clear directive was needed from senior leadership to support this approach. This was described as central to permitting staff to work in this way and staff were clear that this support would need to be active rather than passive, with actions to demonstrate support. Actions of support suggested by staff included clear communication (i.e., making this approach a clear directive), and putting in place the resources and tools needed by staff e.g., changes to estates; frameworks for managing risk; and allowing staff time for training and preparation.

…it would have been beneficial if there’d been a bigger range of staff [at the training] in terms of I guess seniority, having people from different operational modalities come in… it would have really reinforced that message that all rungs of the trust are behind this as an approach. (P2)

I know at my level…everybody is going, “What a good idea,” but I haven’t had any indication that anybody more senior than myself and higher up the hierarchy is … somebody said that the head of CAMHS is up for it but then there’s this huge gap in between that you don’t know how that’s dribbling down and being allowed to happen. Any organisational change, it’s a top down and a bottom up. You can’t just rely on the people on the ground to do it. (P7)

Some staff gave examples of where this approach had worked well: “There was a presentation to the managers. I think that’s what it was, that’s where I heard about it, so I completely bought into it” (P14) and

We have a really good manager here…she did a lot of this stuff when she was working clinically with young people, so she’s always been like, “Yeah, go for it, it sounds great,” and really encouraging of all this sort of approach. (P13)

#### Sub-theme b: harnessing the role of firsthand experience

Previous experience with nature and NBAs was described as playing a crucial role in individual buy-in to this approach because those who had experience were able to anticipate the potential benefits for CYP: “If you believe in, that it’s going to work and want to try it, you can make it work” (P14). This highlights the importance of experiencing the approach as part of training, and utilising firsthand experience service-wide to ensure buy-in and support.

…I think because of my background…I did my forest school training so I’ve always been very pro nature connectedness and getting children especially outside… I think for my own mental health it’s really important for me to be out in nature a lot… if I can take a session outside and it benefits both the child and me, and I can see the benefits of being outside or within those environments, I’d much prefer to have the option to do that. (P5)

…evidence increases motivation doesn’t it…could really I think open people’s eyes to how it can be. I’m just thinking about the work that I’ve done that I’ve offered is because first of all I was a participant and I went to a workshop and I ended up delivering those workshops. But if I hadn’t experienced it myself first I don’t think, yeah, it wouldn’t have happened. (P6)

There were contextual differences which are important to consider in terms of the adoption of NBAs in clinical services. For example, specific units or services practice in different contexts, such as schools or in-patient units which has implications for existing ways of working e.g., exposure to nature-based working, and also to what extent they are used to being adaptable or flexible in their practice e.g., holding sessions outside of clinic rooms routinely. This was described by staff as being linked to buy-in in terms of exposure to more, or less, adaptive, creative, or flexible ways of working and in turn, to strategies such as dynamic risk management.

… there’s also going to be barriers to people who are a bit more, not old-fashioned but a bit stuck in their ways and don’t like change and don’t want to change their practice probably. … I mean, I think for us, because we work in the mental health support team, so we are not clinic-based. We are very much based in schools, so we’re out and about, so we are adapting as we go all the time. (P14)

### Sub-theme c: demonstration of support: action and communication

Staff raised concerns that using NBAs may incur additional resource. Many raised a lack of time for travel, planning, paperwork and risk assessments as a potential issue. The lack of an appropriate location and space was a common response and difficulties with accessibility especially for staff and patients with additional health needs was identified: “I would like to go out and use outdoor/ nature-based spaces for my work with some clients- but generally we cannot access this near to our clinic spaces and we don’t have time to travel or easy access to other venues to work from” (Wave 2, P62). Transport for staff and patients to an outdoor location was a concern as was the impact of the weather: “I feel concerned about how weather could affect it, and if it would mean needing to change locations frequently which could be disruptive” (Wave 1, P11).

Level of buy-in and governance support was felt to be demonstrated through (in)action and communication of senior staff. Staff were looking to those higher up to demonstrate their support for this approach through actions, such as facilitating staff to have time for training and preparation, and communication, i.e., making this approach a clear directive from management: “…maybe the service leader needed to say, “No, actually, we’re taking the day and we’re all going on it.” I guess it’s got to be valued from the top, hasn’t it?” (P14).

Leadership that supported staff to engage with NBAs and gave them permission to work in this way was felt to be important for implementation on the ground.

Further examples of this included access to or provision of appropriate space and resource and protection of time.

Really what should happen is that the Trust should protect space around every clinic that we have every young person’s therapeutic space, and have a green space in there. As much as we need a car park, we need a green space…For me, it should be enshrined at a higher level than even … This programme shouldn’t need to come along and say could we have a garden please, it should be part of the approach from the start. (P2)

These actions were described as going hand in hand with buy-in, in terms of senior management showing this approach is valued, and that staff are valued, and reflecting this in terms of time and resource for practitioners, whether this is administrative, caseload, or in terms of physical resources:

That I would be designated a couple of days by my managers officially to go and engage in a group and support with a group as part of my development as a clinician and part of the development of the service…If you want it to develop, I don’t care if it’s me or not, whoever is involved, for them, they need to be given that space to develop the ideas. (P7)

It was felt that evidence for NBAs as an approach would further motivate this provision of resource and time.

## Discussion

Policymakers and research communities have acknowledged that adopting new forms of information and knowledge can contribute to addressing some of the challenges faced by CAMHS, but the process of adopting and implementing new approaches is less well understood [29]. Nature-based approaches have been identified as a potential way to support not only the health of service users but also the health of the staff who support them. However, how to implement this approach within the setting of CAMHS is not well understood. Our findings suggest multiple barriers to implementation, often in the form of organisational or cultural factors, such as the risk averse nature of the service. Our work also elucidates several potential facilitators which may address or mitigate some of these barriers. These potential enablers, such as harnessing the role of firsthand experience, warrant further exploration in the implementation of NBAs in CAMHS.

The cultural tensions raised in our study align with barriers identified when implementing new practices more broadly within CAMHS, including clinician attitudes and flexibility, team culture and ethos, and safety and risk policies [6, 14, 18]. There were clear concerns from staff that the culture of CAMHS may be restrictive in terms of implementing NBAs. Concerns were voiced about a perceived lack of evidence for NBAs, uncertainty around the form and function of NBAs in CAMHS, and the promotion of risk-taking. This aligns with implementation frameworks which highlight the importance of evidence and stakeholder awareness of evidence [30]. Peters-Corbett and colleagues’ systematic review found both organisational and individual level barriers similar to those described in our study; with a poor level of knowledge of the approach and its benefits, and negative attitudes towards the approach impeding implementation [14]. Our findings build on this, with staff describing how NBAs were perceived to lack empirical evidence or how communication about the evidence base was poor. This perceived lack of empirical evidence may also contribute to fears about how NBAs might be viewed by other staff members, service users or parents/carers, that moving away from traditional approaches may be deemed as not ‘proper work’; making staff reluctant to implement NBAs. Further, participants reflected on the value of different types of evidence when implementing new approaches, with experiential knowledge described by some as particularly important. This aligns with a growing understanding of the value of experiential knowledge in the health literature for informing health services, albeit in this instance from a staff perspective as opposed to a service user perspective [31].

Our findings suggest that approaches such as harnessing the role of firsthand experience and clearer communication from senior leadership could help to mitigate concerns around the evidence base of NBAs. Communication about the evidence behind NBAs and how the approach fits within the CAMHS directive was highlighted as an area which would support staff to implement NBAs, with respondents advocating for consistent and clear communication across all levels of the service. Firsthand experience of the approach was described as central to developing an understanding of the benefits that could be achieved for both staff and service users. Staff felt that experiential knowledge could serve to compliment empirical knowledge, supporting stakeholder buy-in and promoting adoption. Providing experience of NBAs to staff at all levels, clinical and non-clinical, may help to address staff concerns around the evidence base of the approach and may therefore support with buy-in and adoption.

In examining the wider cultural challenges associated with implementing NBAs in mental health services, our findings highlight a significant conflict between a prevailing risk-averse culture and an approach that promotes positive risk-taking. Historically, risk perception in mental health services has been oriented towards risk reduction, leading to norms that can negatively impact decision-making and influence both the treatment and perception of service users [32]. Participants in this study noted that NBAs are often perceived as risky and their implementation would require substantial support, including organisational permission, appropriate tools, resources, training, and dynamic risk assessment processes. Without such support, staff ability to work flexibly, creatively, and in a person-centred manner would be severely impeded [14, 18].

The study participants expressed varied views on risk. While many perceived the risks associated with NBAs as barriers, some recognised risk as a potentially beneficial and normal aspect of working with young people. Case studies have aimed to challenge the definition of individuals with mental health difficulties as inherently ‘risky’ and to promote the value of collaborative decision-making [30]. Despite existing research and policy advocating for shared decision-making, transforming practice around risk in mental health services necessitates a cultural shift. This shift involves endorsing positive risk-taking and leveraging its potential benefits for recovery [33].

Supporting this call for cultural change, participant perspectives on risk were often shaped by their professional backgrounds and experiences in other workplaces. For instance, those with a background in social work exhibited broader views on risk and demonstrated greater openness to managing risk dynamically. Additionally, staff with more experience felt more confident in engaging in positive risk-taking. Effective implementation strategies must therefore consider risk on both cultural and individual levels, aiming to foster more positive perceptions of risk across the service and equip individuals with the confidence to manage risk appropriately [34].

While our interview findings described a cultural tension around implementing NBAs in CAMHS, the survey data provide tentative support to suggest that a cultural shift could be beginning; with ‘permission’ to engage in NBAs perceived not only by those who attended training but across a broader sample of staff. Existing research confirms the importance of staff attitudes and team culture for implementation of NBAs [6] but how this shift might be facilitated is less well understood. Approaches for promoting implementation of NBAs in CAMHS centred on the need for buy-in and governance support, through which many of the cultural barriers stated above could be mitigated.

Buy-in and governance support and involving a range of stakeholders were raised by staff in this study as factors which could support cultural change. In line with research on other interventions in children and young people’s mental health care, the need for buy-in and governance support was highlighted as critical for supporting implementation on the ground [14]. Staff reflected that passive support through training opportunities was not enough. For buy-in across all levels of the service, more active support was sought by staff. Our findings highlight several different ways in which senior leadership could support the implementation of approaches such as protecting staff time for training and preparation; access to space; consistent and clear communication across all levels of the service; providing support in the form of resource (administrative, caseload, or physical) and management of staff capacity to implement new approaches. These actions were described as going hand in hand with buy-in, in terms of senior management showing this approach is valued, and that staff are valued. In turn, these clear actions from senior management may help to promote cultural change and a shift in staff attitudes to NBAs within the CAMHS setting.

Participants described how the cultural change required to support approaches such as NBAs within CAMHS may differ by context, with contextual differences likely to play a significant role in the implementation of NBAs. While existing research indicates additional challenges in settings like schools [16], our findings suggest that the school context may also present unique benefits. The implementation context—whether clinical or non-clinical, such as schools, in-patient units, or CAMHS clinics—affects the extent to which existing practices align with flexible and adaptable working methods, such as holding sessions outside clinic rooms. In environments where staff are already accustomed to working flexibly, NBAs align more closely with their usual practices, making implementation smoother. For instance, in schools, where dynamic risk management and adaptable session locations are more common, staff found NBAs to be more compatible with their routine practices. This underscores the importance of considering the different contexts within CAMHS when implementing NBAs [20].

In contexts where the implementation of NBAs necessitates very different ways of working, the involvement of stakeholders across the service, including service users, parents, clinicians, and management may help to mitigate barriers to implementation. Similarly to other studies of intervention implementation in healthcare settings [14, 18], this was thought to be particularly important for NBAs, which represent a departure from familiar ways of working for clinicians and potentially also to service user expectations. In line with one other study of NBAs in CAMHS [6], activities such as presentations to management and asking for input from CYP were thought to support clinicians when putting NBAs into practice.

### Strengths and limitations

This research has been strengthened by a mixed methods approach which has facilitated capture of a range of perspectives across the trust and enabled a deeper exploration of the topic. The synthesis of survey and interview data provides an understanding at both Trust and individual level. Utilising the survey data to inform our interview topic guide enabled us to delve deeper into how NBAs might work for different individuals in different roles and settings within CAMHS. The collaboration between researchers and clinicians is another strength of this work which has enabled access and insights which would not otherwise have been possible. Our sample may also have been limited to those who were interested in, and therefore potentially more open to, the idea of NBAs, which may have impacted the findings of our study and the largely positive perceptions of NBAs reported. Larger studies across multiple Trusts may be better able to capture a wider range of views and perspectives. The utilisation of unvalidated questions for the assessment of staff knowledge and attitudes towards NBAs in this study is acknowledged as a limitation.

## Conclusion

The implementation of NBAs in mental health service settings for CYP presents both significant challenges and opportunities. Our findings highlight key barriers such as the need for additional time, resources, and organisational support, as well as the pervasive influence of a risk-averse culture. Staff perceptions of risk, shaped by their professional backgrounds and levels of experience, play a crucial role in determining their willingness to engage with NBAs.

Experiential evidence may be pivotal in fostering a deeper understanding and appreciation of the benefits of NBAs. Providing opportunities for staff at all levels to engage directly with these activities can enhance buy-in and support the adoption of NBAs. Moreover, recognising the contextual differences in various implementation settings, such as schools, in-patient units, and CAMHS clinics, is essential. Environments where flexible and adaptable working practices are already established may offer a more conducive atmosphere for NBA integration.

Our study has shown that it is important to cultivate a cultural shift towards positive risk-taking, supported by comprehensive training and dynamic risk management strategies. By focusing on experiential evidence and recognising contextual differences, implementation strategies can be tailored to support staff familiarity and confidence with implementing NBAs which may facilitate more effective and widespread implementation of this approach. Additionally, fostering a supportive workplace culture that values collaborative decision-making and experiential learning may further enhance the integration process. Future research should continue to explore these dynamics, providing a nuanced understanding of how NBAs can be effectively integrated into diverse mental health service settings to improve outcomes for young people.

## Supplementary information

Supplementary Material 1.

## Data Availability

The data that support the findings of this study are available from the corresponding author on reasonable request.
