# Supplementary material for: Nature-based approaches: a mixed methods study of facilitators and barriers to implementation in CAMHS
Source: BMC Health Serv Res. 2024 Nov 8;24:1369. doi: 10.1186/s12913-024-11541-8 (PMC11549852; doi:10.1186/s12913-024-11541-8)
Supplement: Supplementary file 1 — Supplementary Material 1. [file 12913_2024_11541_MOESM1_ESM.docx]

**Topic guide for interviews**

You’ve been selected for an interview because you are work at Cornwall CAMHS. We’d like to ask you some questions about your experience and attitudes towards integrating nature-based approaches (NBAs) in CAMHS.

Do you consent to being interviewed (will have received and completed consent form beforehand, this is to confirm consent) and can we record you? [Interviewer reminds interviewee the content of Information Sheet and Consent form if necessary.]

1. Can you tell me what your involvement has been in CAMHS Goes Wild (CGW)?
   - Prompt – Did interviewee take part in Natural Academy Training (either intro or full course)? If not, have they discussed NBA-s with colleagues who have?
2. What did you think when you first heard about it?
   - Prompt – Were you interested in it or not? Why was that?
3. What do you think now?
   - Prompt – What changed for you?
4. What do you consider to be the outcomes or changes that using NBAs has had for staff?
   - Prompts: job satisfaction; autonomy; creativity; revitalised practice; increased wellbeing; reduced burnout, etc.
   - Can you give me an example?
5. Do you think that the outcomes have been the same for all staff? In what ways have they been different?
6. We are very curious about how NBAs cause outcomes. How do you think the programme has caused, or helped to cause [**one outcome identified by respondent**]?
   - [Then repeat question for **other outcomes identified by respondent**]
7. Do you think the Natural Academy Training has changed the way staff think or feel about NBAs in CAMHS in any way? In what ways? Can you provide examples?
8. What do you think the impact of using NBAs has had for service users? Can you give me an example?
   - Prompt: improved mental health outcomes; additional benefit; increase choice and flexibility; increase personal resourcing
9. Do you think that the outcomes have been the same for all service users? In what ways have they been different?
10. There are lots of ideas about how NBAs work, and we think they probably work differently in different places or for different people.
    - One of those ideas is it gives staff more autonomy to be creative. Has it worked at all like that here or for you? Can you give an example?
    - Another idea is that by being in nature, staff feel more relaxed too and that helps them do a better job. (Immune-mediated and physiological shifts in autonomic nervous system//Physical activity//attention restoration)
    - Another idea is that it increases job satisfaction, has it been like that for you or not?
11. What are the barriers to NBAs working well? What do you think is going to get in the way? Why do you think that? Do you have an example?
12. We’ve heard that NBA work differently in different places, what do you think it is about this place that makes it work so well/less well?
13. If you could change something about this programme to make it work more effectively here, what would you change and why?
14. What else do you think we need to know to really understand how this programme has worked here?

Interviewer thanks interviewee and offers a thank you voucher.
